# Increased Online Aggression During COVID-19 Lockdowns: Two-Stage Study of Deep Text Mining and Difference-in-Differences Analysis

**DOI:** 10.2196/38776

**Published:** 2022-08-09

**Authors:** Jerome Tze-Hou Hsu, Richard Tzong-Han Tsai

**Affiliations:** 1 Center for Geographic Information Science Research Center for Humanities and Social Sciences Academia Sinica Taipei Taiwan; 2 Taipei Municipal Jianguo High School Taipei Taiwan; 3 Department of Computer Science and Information Engineering National Central University Taoyuan Taiwan

**Keywords:** natural language processing, lockdown, online aggression, infoveillance, causal relationship, social media, neural networks, computer, pandemic, COVID-19, emotions, internet, sentiment analysis, Twitter, content analysis, infodemiology

## Abstract

**Background:**

The COVID-19 pandemic caused a critical public health crisis worldwide, and policymakers are using lockdowns to control the virus. However, there has been a noticeable increase in aggressive social behaviors that threaten social stability. Lockdown measures might negatively affect mental health and lead to an increase in aggressive emotions. Discovering the relationship between lockdown and increased aggression is crucial for formulating appropriate policies that address these adverse societal effects. We applied natural language processing (NLP) technology to internet data, so as to investigate the social and emotional impacts of lockdowns.

**Objective:**

This research aimed to understand the relationship between lockdown and increased aggression using NLP technology to analyze the following 3 kinds of aggressive emotions: anger, offensive language, and hate speech, in spatiotemporal ranges of tweets in the United States.

**Methods:**

We conducted a longitudinal internet study of 11,455 Twitter users by analyzing aggressive emotions in 1,281,362 tweets they posted from 2019 to 2020. We selected 3 common aggressive emotions (anger, offensive language, and hate speech) on the internet as the subject of analysis. To detect the emotions in the tweets, we trained a Bidirectional Encoder Representations from Transformers (BERT) model to analyze the percentage of aggressive tweets in every state and every week. Then, we used the difference-in-differences estimation to measure the impact of lockdown status on increasing aggressive tweets. Since most other independent factors that might affect the results, such as seasonal and regional factors, have been ruled out by time and state fixed effects, a significant result in this difference-in-differences analysis can not only indicate a concrete positive correlation but also point to a causal relationship.

**Results:**

In the first 6 months of lockdown in 2020, aggression levels in all users increased compared to the same period in 2019. Notably, users under lockdown demonstrated greater levels of aggression than those not under lockdown. Our difference-in-differences estimation discovered a statistically significant positive correlation between lockdown and increased aggression (anger: *P*=.002, offensive language: *P*<.001, hate speech: *P*=.005). It can be inferred from such results that there exist causal relations.

**Conclusions:**

Understanding the relationship between lockdown and aggression can help policymakers address the personal and societal impacts of lockdown. Applying NLP technology and using big data on social media can provide crucial and timely information for this effort.

## Introduction

### Background

On March 13, 2020, the United States declared a state of emergency in response to the COVID-19 pandemic. Many states imposed lockdown measures to slow down the spread of the virus. However, lockdown (stay-at-home) policies affect many aspects of human life. The frustration and loneliness people experience under extended periods of confinement may predictably have negative psychological impacts [1-3]. Furthermore, frustration can manifest itself through increased aggressiveness [4]. In a time when people live closely beside intimate family members, emotional problems, such as suicidal thoughts and aggressiveness, may lead to destructive behaviors and have an immediate impact on society [5,6]. Whether scientific investigations corroborate such observations can have significant policy implications for public or private governance. Unsurprisingly, the relationship between lockdown and adverse psychological effects has attracted increasing attention from multiple disciplines of researchers. However, there have been few robust tests of the causal relationship between lockdown and aggressive emotions. This research used machine learning to produce robust data. Then, we used a statistical difference-in-differences analysis to estimate the causal relationship between lockdown and increased online aggression. The application of machine learning technologies in social science research can provide new information in a much broader scope at a much higher speed.

### Related Works

#### Negative Impacts of Lockdown

At the individual level, studies have shown that lockdown is associated with suicidal ideation, anxiety disorder, nightmares, depression, loneliness, and poor mental health [7-12]. At the societal level, a lockdown’s adverse effects are manifested through significant increases in divorces, sexual violence [13], and domestic violence [14]. All these effects pose considerable threats to the stability and well-being of individuals and society. Therefore, it is an urgent task to understand these harmful actions under COVID-19 lockdowns.

Other research in psychology has focused on the deterioration of mental health before and under lockdown [7]. The authors observed an increase in certain health behaviors 1 month into lockdown by comparing prelockdown and postlockdown survey data. However, the authors did not analyze the causal relationship between lockdown and these behaviors.

Emotion is one of the main drivers of human action. It is reasonable that a more aggressive state of mind leads to aggressive behaviors like domestic and sexual violence. The influential frustration-aggression theory [15] suggests that aggressive behavior results from frustration caused by thwarting individual goals. In the early months of the pandemic, lockdown led to many canceled plans and unaccomplished goals. Therefore, a causal relationship between lockdown and increased aggression is reasonable.

Notably, a study investigated the correlation between lockdown and increased aggression [2]. Killgore et al conducted a questionnaire survey [2]. They used the Buss Perry Aggression Questionnaire to measure aggression levels in patients under and not under lockdown during the initial months of the pandemic in the United States. They found a statistically significant increase in the following 4 kinds of aggression between lockdown and nonlockdown groups: physical aggression, verbal aggression, anger, and hostility. However, owing to limitations in traditional questionnaire methods, such as the lack of data before the pandemic, this research could not assess the causal relationship between lockdown and increased aggression. Furthermore, because of practical limitations in survey administration, the authors had to survey different participants in every sampling, which provides an additional source of uncertainty.

#### COVID-19 Twitter Sentiment Analysis

Online analysis of tweets using natural language processing (NLP) has provided valuable information in health-related research. General sentiment analysis has been performed to examine people’s emotions under lockdown [16,17].

Some are related to specific topics, such as vaccination [18-20], while others are related to specific regions or countries [21]. However, most studies did not analyze the relationship between lockdown and emotions to the best of our knowledge. Su et al [22] analyzed the psycholinguistic features in 2 different cities going into lockdown. While this can capture specific rising trends in tweet words, the lexicon frequency analysis method does not capture each word’s context. Thus, it cannot predict emotions as accurately as neural network models [23].

### Our Study

This paper addresses the weaknesses of the current psychology and NLP research. Most of the recent literature in psychology has not offered meaningful evidence to the causality between lockdown and aggressive emotions. On the other hand, current NLP research in sentiment analysis mainly focuses on optimizing methods for machines to capture emotions in vast volumes of digitalized human discourse. However, the current body of NLP literature in the public health area rarely probes into causal relations of social phenomena.

We used new NLP technology to conduct a virtual longitudinal study of online Twitter users and their tweets to investigate the impacts of lockdowns on the following 3 kinds of aggression: anger, offensive language (offensive), and hate speech (hate). Our infoveillance method allows us to discover trends in aggression levels that can provide important information for policy makers and health professionals. Moreover, data before and after lockdown allows us to estimate the potential causal relationship between lockdown and increased aggression using the difference-in-differences analysis, an established econometric method to understand the causal relationship in nonexperimental time-series data [24]. This interdisciplinary method yields robust results in understanding the relationship between lockdown and increased aggression, and it opens up new potentials for applying NLP and internet technology to support medical research.

## Methods

### Overview

First, we sampled a group of Twitter users across the United States as our subjects of analysis. Then, we used Twitter’s application programming interface to obtain all the tweets the sampled users posted between January 1, 2019, and October 1, 2020. Our objective was to use a neural network model to detect different levels of aggressive emotions during different periods in these tweets. We selected the Bidirectional Encoder Representations from Transformers (BERT) model, a state-of-the-art language model that can understand the meanings of emotions through contexts and nuances better than previous lexicon-based models [23,25]. To train the BERT model for emotion detection, we collected training data based on established data sets [26-28]. Afterward, we classified the tweet emotions using our trained BERT model. We conducted an observational analysis to compare and contrast aggression levels between different geographies, lockdown statuses, and times. After observing an increase in aggression after lockdown, we measured the relationship between lockdown status and aggression levels using the Poisson regression as a difference-in-differences estimation.

### Twitter Data Sampling

In the United States, states retain the power to implement lockdown policies. Therefore, this research used the state lockdown status to determine whether an individual user was under lockdown at a particular time. We randomly sampled Twitter users geographically tagged with the states in the United States as our longitudinal internet study participants. After sampling the users, we sampled every tweet they posted in 2019 and the first 6 months after President Trump declared a national emergency in March 2020. Our sampling yielded a spatiotemporal data set of 1,281,362 tweets posted from January 1, 2019, to October 1, 2020, by 11,455 Twitter users. The sampled users came from all across the United States, including users from all 50 states. In this study, we used these tweets to investigate the relationship between lockdown and social media aggressiveness. All tweets followed a data preprocessing protocol [29] before being analyzed by the BERT model for emotion detection.

### Training Data Collection

In order to detect aggressive emotions in the tweets, we trained a BERT neural network binary classification model for each of the 3 aggressive emotions. For each model, we collected different training data sets. Our definition of each emotion is identical to that of the training data set. Table 1 contains the definitions for the 3 aggressive emotions, with Table 2 providing sample text for each.

**Table 1:** Definition for each emotion.

Aggressive emotion	Definition
Anger	A strong feeling of displeasure or antagonism [26]
Offensive language	Speech that contains unacceptable language (profanity) and is potentially harmful to a disadvantaged group [27]
Hate speech	Language that expresses hatred toward a targeted group and is intended to be derogatory, insulting, and humiliating [27,28]

**Table 2:** Sample text containing each aggressive emotion.

Aggressive emotion	Sample text
Anger	I hope this all ends soon. This is hell
Offensive language	Are people really this stupid?
Hate speech	@user The rot starts from the top......Trumps wankers are all racist......F*ck them all!

#### Anger

We selected the GoEmotions data set to train the anger classification model [26]. It is one of the largest manually annotated data sets of 58,000 English Reddit comments. In the data set, each sentence is annotated to identify the presence of 28 relatively common emotions. To train the BERT model for binary classification, we selected the 6000 sentences that contain anger and a random sample of 6000 other sentences that do not contain anger. We selected 1000 other annotated comments for testing. Based on GoEmotions, anger is defined as “A strong feeling of displeasure or antagonism.”

#### Offensive Language

Offensive language is speech that contains unacceptable language (profanity) and is potentially harmful to a disadvantaged group. We selected the “Automated Hate Speech Detection and the Problem of Offensive Language” (AHSD) [27] data set as our training data set, which contains 24,802 human-labeled tweets. We randomly sampled 7750 sentences for training and 613 for testing. The study distinguishes hate speech with real harmful intentions from general offensive lexicons. For example, many teenagers often use terms like f*ck and b*tch in a casual manner that does not intend harm. AHSD provides annotated data for offensive language and more harmful hate speech.

#### Hate Speech

Unlike general offensive language, hate speech is a more specific language that causes intentional harm. To train our hate model, we merged the AHSD data set, as mentioned earlier, with the Large Scale Crowd Sourcing [28] data set, which provides an additional 2067 tweets labeled hateful by humans. We randomly sampled 6450 sentences for training and 639 for testing. Both of these data sets identify hate speech as the language that expresses hatred toward a targeted group and is intended to be derogatory, insulting, and humiliating. This definition has been widely used in previous research [30-32].

### Model Training

We used the BERT model [29] to identify emotions in tweets. This pretrained neural network model is one of the most powerful models in emotion understanding. With its abundant pretraining data from the entire English Wikipedia, the model already had a basic understanding of the English language before we conducted the final fine-tuning. The model’s contextual embedding allows it to understand words regarding context, taking its language understanding ability beyond traditional lexicon analysis. Our model architecture is constructed under python modules pytorch 1.8.1 and transformer 4.11.0. Using the training data, we obtained great performing models for all 3 of our target emotions (specific statistics are shown below).

### Model Evaluation

**Figure 1:**
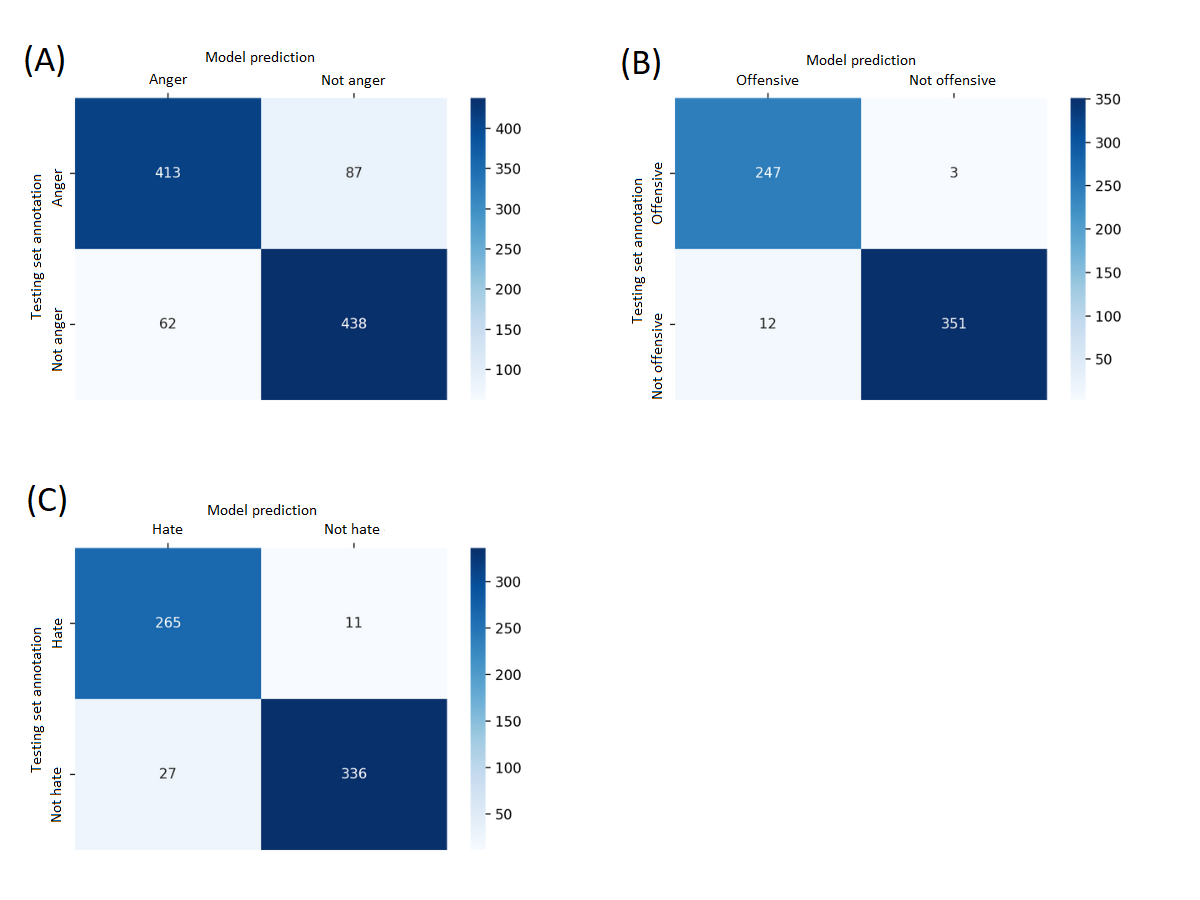
Confusion matrices for our models: anger (A), offensive (B), and hate (C). The bottom-right and top-left quadrants are where the models predicted correctly, which represent true negatives and true positives, respectively. A darker quadrant color indicates greater prediction.

We first tested our model predictions on the testing set (train-test split). The results are shown in Table 3, along with the confusion matrices in Figure 1. Then, we tested our model on the sampled Twitter data set used for further analysis. To evaluate the model performance on our self-sampled Twitter data, we selected 1080 tweets, 540 from people under lockdown and 540 from people not under lockdown, with 5-6 tweets randomly selected from each week. Then, 2 native English speakers annotated the tweets based on the definition for each emotion above. The Cohen kappa values between annotators and our model’s performance are reported in Tables 4 and 5, respectively. Through this, we can validate our model competence on Twitter data used in further down-stream analysis.

**Table 3:** Model performance on the testing set.

Model	Precision	Recall	F1
Anger	0.869	0.826	0.847
Offensive language	0.953	0.988	0.970
Hate speech	0.956	0.920	0.933

**Table 4:** Cohen kappa interrater agreement between the raters.

Emotion	Kappa
Anger	0.928
Offensive language	0.937
Hate speech	0.890

**Table 5:** Model performance on the sampled Twitter data set.

Model	Precision	Recall	F1
Anger	0.795	0.888	0.839
Offensive language	0.843	0.922	0.880
Hate speech	0.810	0.872	0.839

### Data Analysis Methods

#### Overview

To understand aggression levels in tweets, we measured the proportion of tweets that contain aggression among all randomly sampled tweets. First, we used separate BERT models for each aggressive emotion (ie, anger, offensive, and hate) to analyze our sampled Twitter data. The analysis resulted in 3 data sets, one for each aggressive emotion. In each data set, for each of the 50 states, we calculated the percentage of tweets containing the aggressive emotion for the 92 weeks from January 2019 to October 2020. This analysis resulted in 3 data sets with 4600 data points each.

Although our data consist of aggressive tweet counts in different spatiotemporal settings, we analyzed the proportion of aggressive tweets among total tweets, rather than the count of aggressive tweets, to investigate the aggression level on Twitter. This is because an increase in aggressive tweet counts may be due to an increase in total tweets posted, which does not necessarily indicate a higher level of aggression. Measuring the proportion of aggressive tweets more accurately depicts the aggression level on Twitter.

Over 3 stages of observation and analysis, we looked at the data from different perspectives. In the first part, we compared aggression levels between groups of different lockdown statuses in the first 6 months of the pandemic. After that, we focused on the states that had undergone lockdown, and we looked at their aggression levels before and after lockdown. Finally, we used difference-in-differences analysis to estimate the impact of lockdown on the increase of aggression.

#### Observing the Difference in Aggression Levels Between Groups Under and Not Under Lockdown

To understand the impact of lockdown on aggressive emotions, we investigated the aggressive tweet proportions for each specific time and location, and compared the proportions in people under and not under lockdown. For that purpose, we designed our first objective. For each week in the 92 weeks from January 1, 2019, to October 1, 2020, we separated states under lockdown from those not under lockdown into 2 groups. Then, we separately aggregated the number of aggressive tweets and total tweets. We calculated the aggressive tweet percentage for each of the 2 groups every week for 92 weeks based on the combined data. Note that the users in each state represented the patients under lockdown and not under lockdown based on the state’s current lockdown status.

#### Observing Aggression Trends in States That Had Undergone Lockdown From the Weeks Before and After Lockdown

In the previous section, we observed and compared aggressive emotions between groups under and not under lockdown. In this section, we focused on understanding the trends in states that had undergone lockdown. More specifically, we looked at the increase in aggression after lockdown by comparing data before and after lockdown. We chronologically aligned the data in each lockdown state based on the initial week of lockdown. More specifically, for every state that had ever undergone lockdown, the week that lockdown started was denoted as week 0. Other weeks were numbered accordingly (ie, the first week after week 0 was week 1, the week before week 0 was week −1, and so forth). Using this method, we visualized the increase in aggression after the lockdown. Note that this was solely an observation of aggression trends before and after lockdown. It did not measure the net impact of lockdown status on aggression levels. To specifically measure the impact and investigate the causal relationship, we applied the difference-in-differences estimation in the next section to quantify the difference in aggression levels between the lockdown and nonlockdown groups in a statistical manner.

#### Difference in Differences Using Poisson Regression

The traditional way to investigate a causal relationship is an experiment conducted on randomly assigned subjects, in which participants are randomly separated into 2 groups. One group receives treatment, and the other does not. However, in many cases, including ours, an experiment is not viable owing to practical or ethical reasons. For example, we cannot randomly assign people and put them under lockdown for an extended time. Some social scientific researchers use multivariate regression to solve this problem, when the independent variable of interest, *X*, and other correlated variables, *Z_1_*, …, *Z_k_*, act together to determine the outcome, *Y*. Although this method can control for the effect of the selected *Z* variables, some other potentially relevant variables might be lacking in data or difficult to identify, leaving a possibility that important variables are not considered. To address this problem, scientists have used the difference-in-differences method.

As Callaway and Sant’Anna indicated, “Difference in differences (DID) has become one of the most popular research designs used to evaluate causal effects in policy interventions” [33]. Difference in differences compares the difference between the treatment group and the control group at a particular time (T1) with that between them at another time (T2), with the 2 times separated by a particular intervention. This method compares the difference at T1 (*D*_T1_) with the difference at T2 (*D*_T2_) and measures whether the difference between *D*_T1_ and *D*_T2_ (difference in differences) has causal relations with the intervention. In short, difference in differences measures whether the intervention causally impacts the difference between *D*_T1_ and *D*_T2_ [34]. In this research, the treatment group refers to those Twitter users under lockdown, and the control group refers to those not under lockdown. The intervention is lockdown. Our objective was to compare the difference in the level of aggression between these 2 groups (*D*_T2_) with that between the 2 groups before lockdown (*D*_T1_) and measure whether the difference between *D*_T1_ and *D*_T2_ is causally related to lockdown.

To implement the difference-in-differences estimation in time-series data, we use fixed effect models. Fixed effect models address unseen variables by controlling for the average in each geographic and temporal data group (data group, in short). The average in each data group is constituted by many factors, including those Z variables that we may or may not know. In other words, the effects of the Z variables we need to control are captured in the average of each data group. By subtracting the average from the outcome in each data group, fixed effect models control for the influence of miscellaneous Z variables and measure the net increase in the Y variable to which the X variable contributes. Taking “fixed effect of the states” as an example, the difference in aggression levels caused by different political tendencies and racial compositions was captured in the average aggression level in each state. After subtracting the average, we now measured how aggression levels increased with respect to each state’s norm. Models with fixed effects used in this research “come closer than does ordinary regression analysis to achieving unbiased estimates of causal effect” [35].

To implement the fixed effect model above, we needed to add the fixed effects to a regression model best suited for our data. Because our original observation was the number of aggressive tweets posted in a specific spatiotemporal setting, our data represented a type of count data. Therefore, we selected the classic Poisson model for count data with fixed effects [36]. The following equation was initially used:

ln(*Aggressive Tweet Count_s,t_*) = *α*_0_ + *α*_1_
*under lockdown* + *μ_state_* + *σ_time_* + *ε*
** (1)**

In this case, *Aggressive Tweet Count_s,t_* is the number of aggressive tweets in a specific state (s) under a specific time (t). *α*_0_ is the constant in standard regression models. Variable *α*_1_ signifies the treatment effect of *under lockdown* on the aggressive tweet count. *Under lockdown* is a binary variable (the explanatory variable in this experiment) that has a value of 0 for not under lockdown and 1 for under lockdown. The model also included state and time fixed effects as follows: *μ_state_* and *σ_time_*. These 2 variables do not have a specific range but rather represent the average of their corresponding groups of data (eg, data in a state or in a specific week). *ε* is the error term included in all statistical regressions.

As aforementioned, however, an increase in aggressive tweet counts may be due to an increase in the total tweets posted, which does not necessarily indicate a higher level of aggression. Measuring the proportion of aggressive tweets more accurately depicts the aggression level on Twitter. Therefore, to measure the proportion of aggressive tweets using this count-based model, we exposed the estimation to the total number of tweets by adding the term ln(*Total*), with the coefficient fixed to 1, to the equation. This action is designed for situations like ours and is supported by the *exposure()* option in Stata 17 software [37]. More specifically, our equation was now as follows:

ln(*Aggressive Tweet Count_s,t_*) = *α*_0_ + *α*_1_
*under lockdown* + *μ_state_* + *σ_time_* + *ε* + ln(*Total_s,t_*) ** (2)**

To understand the mechanism of how adding ln(*Total_s,t_*) allows us to estimate the proportion rather than the count, we can look at the equation in the following way: when we subtract ln(*Total_s,t_*) on both sides of the equation, the estimation is equivalent to modeling the proportion of aggressive tweets. The equation is as follows:

ln(*Aggressive Tweet Count_s,t_* / *Total_s,t_*) = *α*_0_ + *α*_1_
*under lockdown* + *μ_state_* + *σ_time_* + *ε*
** (3)**

Our model comes closer to capturing the unbiased causal effect of the independent variable of interest on the dependent variable in observational data [35]. Since most other independent factors that might affect the result, such as seasonal and regional factors, have been absorbed by time and state fixed effects, a significant result in this difference-in-differences analysis can not only indicate a concrete positive correlation but also strongly suggest a causal relationship. All analyses were conducted using Stata BE Edition 17.0 (StataCorp).

## Results

### Observing the Difference in Aggression Levels Between Groups Under and Not Under Lockdown

**Figure 2:**
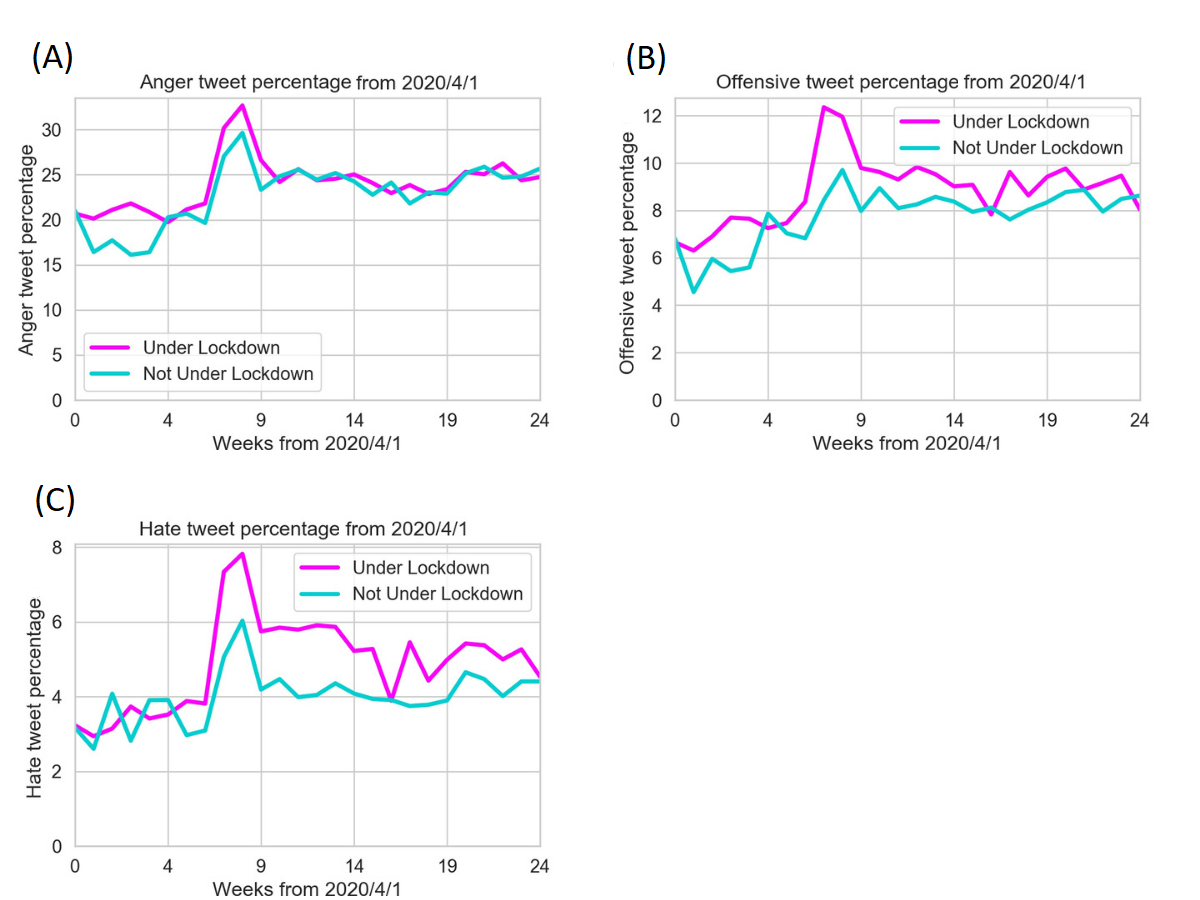
Weekly aggressive tweet percentages since April 1, 2020, for all 3 aggressive emotions: anger (A), offensive (B), and hate (C). States under lockdown (magenta) and not under lockdown (cyan) are indicated.

**Figure 3:**
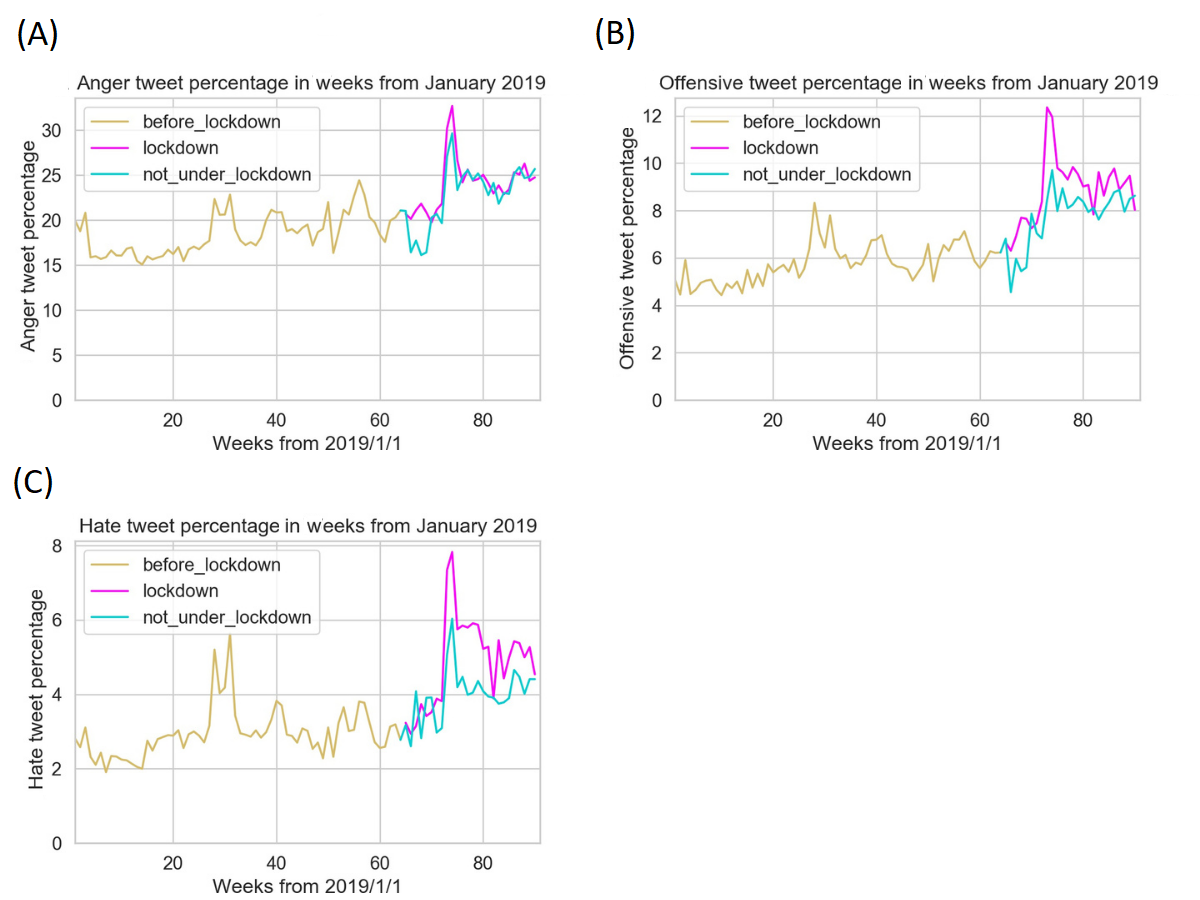
Weekly aggressive tweet percentages since January 1, 2019, for all 3 aggressive emotions: anger (A), offensive (B), and hate (C). States under lockdown (magenta), states not under lockdown (cyan), and data before any lockdown started (yellow) are indicated.

This analysis compared aggression levels between states under and not under lockdown. The United States declared a state of emergency on March 13, 2020. Among 42 states that had ever imposed lockdown, 40 started lockdown in the 2 weeks between March 20 and April 4, 2020. Figure 2 shows data from April to October 2020, when the pandemic was getting severe in the United States and some states began lockdown. It illustrates the weekly difference in aggression levels between groups under and not under lockdown. Figure 3 zooms out the timeframe to include data from 2019, putting data under the pandemic into a broader perspective.

#### Anger

In terms of the intense feeling of displeasure or antagonism, there was a sudden increase in tweet count in May 2020, particularly evident in those under lockdown. In the first 9 weeks since April, average anger levels were 2%-3% higher in the group under lockdown than in the other group. Figure 3 shows that the percentage of angry tweets fluctuated around 20% in 2019. Coming into May 2020, the percentage rose to as high as 34%. In the summer of 2020, anger levels decreased, and the 2 groups demonstrated similar angry tweet percentages.

#### Offensive Language

For unacceptable language that can potentially harm a disadvantaged group, tweet proportions increased sharply for 7 weeks since April 2020 (Figure 2). Offensive levels fluctuated around 6% in 2019 (Figure 3). In the 7th week, offensive tweet percentages in under and not under lockdown groups surged to 12.2% and 9.8%, respectively. Afterward, the numbers started to gradually decrease. Although similar trends were seen in both the under and not under lockdown groups, the tweet percentages under lockdown were consistently 2%-3% higher than the values in the other group.

#### Hate Speech

For derogatory, humiliating, and insulting speech intended to express hatred to a targeted group, tweet proportions reached the peak in the 8th week from April 1, 2020 (Figure 2). Hate speech percentages surged from around 3% when the pandemic started to 8% and 6% for people under and not under lockdown. Percentages decreased in the summer of 2020, gradually stabilizing in the months thereafter. Similar to trends in offensive language, hate tweet percentages were considerably greater than the values in 2019 after the decrease (Figure 3). After the initial surge, hate speech percentages under lockdown were 1%-2% higher than the values in the other group.

Aggression levels surged in all 3 kinds of aggressive emotions, reaching the peak around 6-8 weeks from April 1, 2020 (Figure 2). People under lockdown demonstrated a more aggressive tendency throughout the process than those not under lockdown. People who were not under lockdown experienced similar trends to those under lockdown, but to a less drastic degree. After the initial peak of increased aggression, all 3 kinds of emotional tweet percentages decreased to a relatively stable plateau. This stabilization might suggest that people are gradually getting used to the situation, and emotions are relatively eased compared with the sudden displeasure in the early days of lockdown. Despite the temporary decrease, aggressive tweet percentages were considerably higher than the values in 2019 (Figure 3).

### Observing Aggression Trends From the Weeks Before and After Lockdown

**Figure 4:**
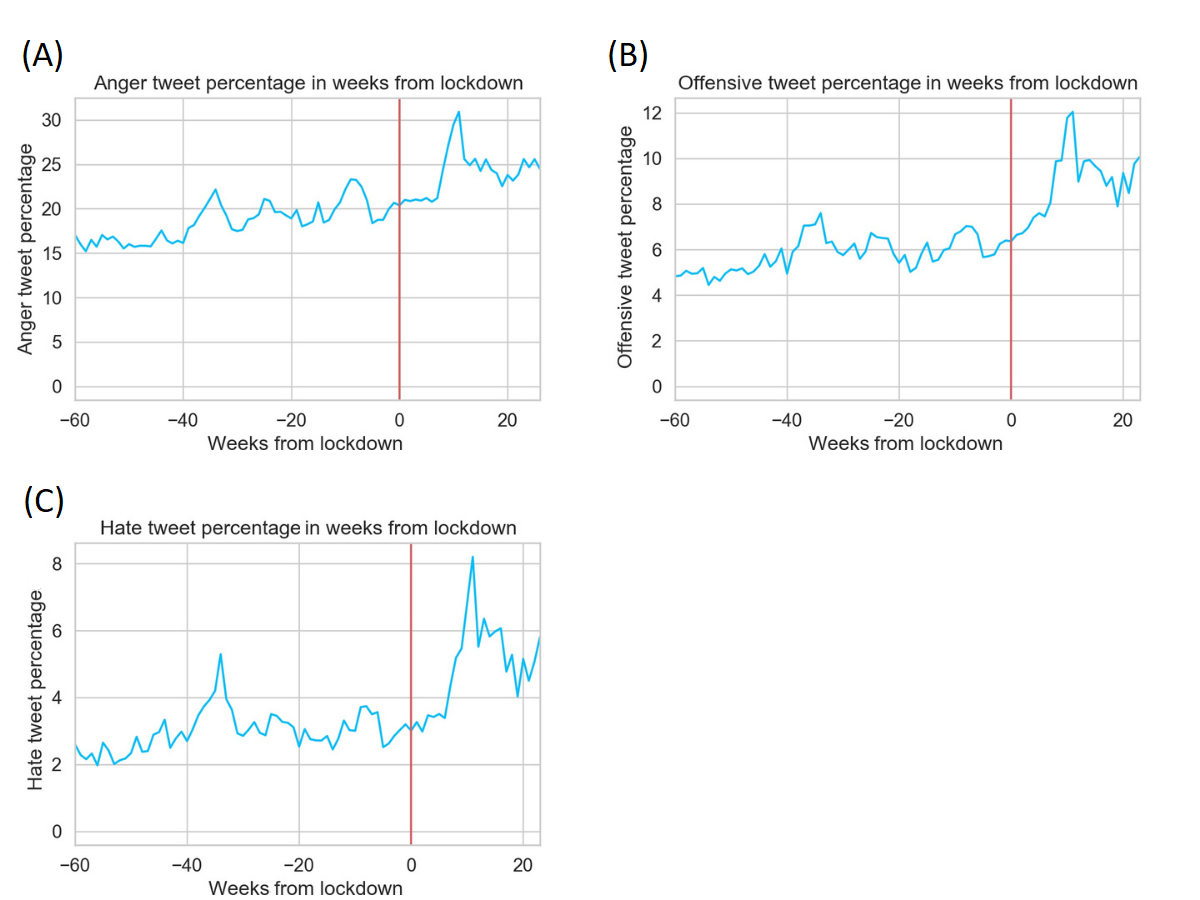
Aggressive tweet percentages for anger (A), offensive (B), and hate (C) before and after lockdown. The vertical red line at week 0 denotes the start of lockdown. Note that because states might have started lockdown at different times, week 0 can differ in different states. Nevertheless, states generally started their lockdown between March 20, 2020, and April 4, 2020.

We selected states that had undergone lockdown and compared their aggression levels before and after lockdown. We visualized the increasing trends of aggression after lockdown. Figure 4 shows the weekly changes in tweets containing the target emotions. In all 3 kinds of aggressive emotions, there was a visible surge in tweet percentage within 10 weeks after lockdown. The table below shows the average weekly tweet percentages (60 weeks before lockdown and 22 weeks after lockdown). In all 3 emotions, the percentage rose after lockdown (anger, 18.51% to 23.77%; offensive, 5.80% to 8.79%; hate, 2.97% to 4.85%). These descriptive data give us a basic grasp of the potential connection between lockdown and increased aggression. Note that this part does not conclude the increase is totally caused by lockdown but rather shows the general trends of aggression before and after lockdown that might be caused by multiple factors. In the next part, we conducted a difference-in-differences analysis to precisely estimate the net impact of lockdown on the increase of aggression.

### Difference in Differences Using Poisson Regression

In this section, we conducted a difference-in-differences analysis using a Poisson regression model. Lockdown was associated with an increase in aggressive tweet proportions for all 3 kinds of aggressive emotions. In a log-linear model, the original coefficient, *α*_1_, between X and Y denotes the increase of ln(Y) for every unit increase of X, which is difficult to interpret due to the presence of logarithm. The incidence rate ratio (IRR) is the exponentiated coefficient of the independent variable of interest, e*^α^*^1^, that demonstrates the increase of Y for every unit increase of X. For all 3 aggressive emotions, the IRR between the aggressive tweet proportion and lockdown status was greater than 1 (anger, 1.049; offensive, 1.168; hate, 1.114), indicating that after the initial lockdown, there were on average 4.9%, 16.8%, and 11.4% increases in emotional tweets for anger, offensive, and hate, respectively. All 3 of the results demonstrated high statistical significance (anger: *P*=.002, offensive: *P*<.001, hate: *P*=.005). Difference-in-differences results are shown in Table 6. Under the control of state and time fixed effects, most possible factors that can lead to misinterpretation were nullified. Therefore, we could measure the net impact of lockdown status on aggression levels. Our estimation strongly suggested a causal relationship between lockdown and increased aggression in all 3 categories of aggressive emotions.

**Table 6:** Results of Poisson regression for emotional tweet proportion and lockdown status.

Lockdown status	Incidence rate ratio	Standard error	*z*	*P* value	95% CI
Under lockdown (anger)	1.049745	0.0163949	3.11	.002	1.018099-1.082375
Under lockdown (offensive)	1.168261	0.0319202	5.69	<.001^a^	1.107345-1.232529
Under lockdown (hate)	1.114432	0.0432653	2.79	.005	1.032780-1.202541

^a^STATA regression yielded *P*=.000.

## Discussion

### Principal Findings

#### Infoveillance Study on Aggressive Emotions Under Lockdown

Understanding the trends of aggressive emotions is the first step to understanding various social problems associated with aggressive behaviors during the pandemic. Inspired by the questionnaire study by Killgore et al [2], we used NLP as an infoveillance method to observe the trends of online aggression in the first few months of the pandemic. We hope this method can support traditional psychology surveys by utilizing computer technology to provide a more efficient way of understanding crowd emotions.

By using statewide lockdown status to analyze tweets, we can capture the peaks and valleys of aggression levels throughout a prolonged time period. We can also identify the difference in aggression levels between groups with different lockdown statuses. There were a few particularly noticeable peaks in aggression levels in the observed timeframe. These aberrations might be able to explain the effects of various social events on public sentiment. From the start of April 2020, when most states imposed lockdown, to the second half of May in the same year, aggression levels rose by a magnitude not found in 2019, with people under lockdown demonstrating a more acute rise than others. As the nationwide deaths from COVID-19 skyrocketed from below 100 per day in late March to over 2000 in mid-April, emotions and lifestyles were impacted unprecedentedly. Through lockdowns, death tolls steadily decreased in the next few months, reaching a lower equilibrium in June. The peaks of all 3 kinds of aggressive emotions were observed in the most severe month of the nationwide pandemic when states were experiencing high death tolls and civil unrest.

After a roughly 2-month period of lockdown, aggression levels reduced. This drop might be due to pandemic fatigue, making people feel less stressed and demonstrating less aggression [38]. According to a report by the World Health Organization, “At the beginning of a crisis, most *people* are able to tap into their surge capacity – a collection of mental and physical adaptive systems that humans draw on for short-term survival in acutely stressful situations. However, when dire circumstances drag on, they have to adopt a different style of coping, and fatigue and demotivation may be the result” [39]. Despite the decrease, those under lockdown still demonstrated a higher aggression level than those not under lockdown.

Although aggression trends roughly follow the same pattern in all 3 kinds of aggressive emotions, each has some slightly different characteristics that reveal the uniqueness of each emotion. Anger was the most common emotion among the 3 emotions. After the initial peak, the aggression lines between groups of different lockdown statuses intertwined in the next few months. Offensive and hate were seen less often than anger. However, offensive and hate levels among those under lockdown were consistently higher than the levels among those not under lockdown in the first 6 months of the pandemic.

Our infoveillance study captured the fluctuation of people’s emotions over a specific timeframe, providing vital information for policymakers and public health professionals.

#### Discovery of a Causal Relationship Between Lockdown and Increased Aggression

Our estimation suggested a causal relationship between lockdown and increased aggression. The Poisson regression analysis designated for count data is suited to estimate the number of aggressive tweets posted in a time period. Time and state fixed effects are able to address the undesired effects of factors other than lockdown status on the outcome. Using this rigorous statistical model, we can show the net impact of lockdown status on the increase of aggressive tweets. The highly significant results in all 3 kinds of aggressive emotions (anger: *P*=.002, offensive: *P*<.001, hate: *P*=.005) matched with the observation in our infoveillance study, that is, people under lockdown have higher aggression levels. Aggressive emotions under lockdown can cause social problems such as domestic violence and divorce. Our findings provide essential information for understanding the causes of aggressive emotions during the pandemic.

#### Potential Policy Implications

Statistics and scientific evidence play crucial roles in rational policymaking during the pandemic [40]. Using big data to detect potential causal relations between lockdown and aggression may guide governments to implement mental health support policies during lockdowns. In the past, mental health support has come in various different forms, including but not limited to domestic violence protection [41], school counseling [42], and psychological consulting [43]. We hope that our spatiotemporal detection of aggression trends may facilitate more efficient allocation of public resources to areas that are most in need. Moreover, we hope to inspire future researchers to use machine learning to detect social trends that invite proper policy responses. Moreover, we hope our causal analysis can raise social and political awareness of the importance of mental health policies during the pandemic.

### Comparison With Prior Work

Killgore et al [2] discovered an increase in aggression levels after lockdown that was particularly evident among those under lockdown. Our research used the Poisson regression model with fixed effects to precisely measure the net impact of lockdown and aggression. This widely established econometric method points to a causal relationship between lockdown and increased aggression [33]. Compared with traditional questionnaire surveys that can only collect data for 1 subject at a time, our data from Twitter are much more versatile. They can be used in other subject research by adjusting the analysis method.

Previous NLP sentiment analysis studies focused on using machines to understand emotions in vast volumes of text [16,17]. However, few of them applied this technology to investigate causal relations of social phenomena in the public health area. Inspired by traditional questionnaire research in psychology, we applied NLP technology to a longitudinal internet study of emotions. This interdisciplinary effort provides crucial information to understand the factors contributing to increased aggression. It opens up new opportunities for NLP technology to make psychology and public health research efficient and timely.

### Limitations and Future Work

Our research has several limitations as well as potential for improvement in future work. First, Twitter data overrepresent younger users who have better access to mobile apps and live in a culture that promotes social media. Such users might not accurately reflect the whole population, as certain groups of different demographic and socioeconomic statuses might be underrepresented [44,45]. However, this limitation is not unique to this study but is present in all studies involving Twitter data. Second, due to time and computational limits, the number of tweets we sampled was not very large considering the total number of available tweets. In the future, we can use this research procedure with an increased number of tweet samples to detect aggression levels in space and time at a more granular scale. Nevertheless, the current number of sampled tweets was sufficient for this research to show aggression fluctuations at the statewide scale and draw statistically significant claims. Third, to determine the lockdown status of each user, this research could only use the lockdown status of the geo-tagged US state of the user. Since we were unable to ask the users about their lockdown status at a personal level, this might have led to some inaccuracies in determining the lockdown status of users. This limitation is inherent to social event studies on the internet. Therefore, our study is conducted under the assumption that users followed the lockdown policies in their state. Furthermore, different types and stringencies of lockdown policies emerged in response to the rapidly changing pandemic circumstances. This research only measured the initial lockdown where people were restricted to staying at home. Future research is open to measuring the effect of lockdown policies at different levels and nuances on aggressive emotions and behaviors. Another source of uncertainty comes from the Twitter user location labels, since the location is per user rather than per tweet (“a user who moves from one city to another [and updates his location] will have all of his tweets considered as being from the latter location” [46]). Moreover, the studies by Gore et al and Frank et al showed that the sentiment of a tweet is highly correlated with the geographical area (ie, city) it was composed in [47,48]. Finally, our research method is not restricted to measuring aggressive emotions. Future research can easily apply our methodology to other emotions and research topics. Using NLP technology to help psychology and public health research has vast potential in the future.

### Conclusions

Infoveillance studies can be immensely useful in the modern world. With recent advancements in NLP, models can be trained to accurately understand emotions in text. NLP technology can be applied to analyze emotions in large volumes of social media data. This large-scale spatiotemporal data of public emotions can be further analyzed to investigate the correlations and causal relations between emotional trends and certain policies like lockdowns. Applying computer technology to social scientific research has vast potential in the future.

## Abbreviations

AHSD: Automated Hate Speech Detection and the Problem of Offensive Language
BERT: Bidirectional Encoder Representations from Transformers
IRR: incidence rate ratio
NLP: natural language processing

## Data Availability

The source code for this study is available on GitHub [49].
